# Aortic valve-sparing variants are getting closer

**DOI:** 10.1016/j.xjtc.2023.12.003

**Published:** 2023-12-19

**Authors:** Marek J. Jasinski

**Affiliations:** a Clinical Department of Cardiac Surgery, Wroclaw Medical University, Wroclaw, Poland; b Royal Brompton and Harefield Hospital, Cardiothoracic Surgery, London, United Kingdom

To the Editor:

**Figure:**
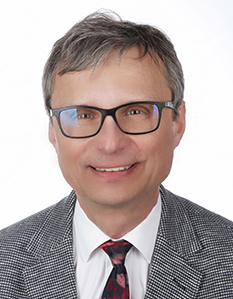

Interest in valve-sparing operations is undoubtedly ongoing, as illustrated by recent articles in AATS journals: *JTCVS Techniques*,^1^ *Operative Techniques in Thoracic and Cardiovascular Surgery*,^2^ and the *Journal of Thoracic and Cardiovascular Surgery*.^3^ A recent meta-analysis confirmed that valve-sparing aortic root replacement with reimplantation is associated with better overall survival and lower risk of need for reintervention compared with valve-sparing aortic root replacement with remodeling. Regarding overall survival, we observed a time-varying effect that favored the reimplantation technique up to 4 years of follow-up, but not beyond this time point, apparently losing the early benefit of Dacron prosthesis stabilization.^4^ Moreover, the results are not homogenous across the different phenotypes with bicuspid aortic valve as a risk factor shown recently by Sharma and colleagues.^3^ There are several issues related to aortic valve reimplantation that may be responsible. They are focusing on the importance of stabilizing the virtual basal ring (VBR) and the ventriculo-aortic junction (VAJ) at the same time. The observations include inconsistent interference between the VAJ and the Dacron prosthesis along the annular circumference and pseudoaneurysm formation risk,^5^ a significant tilt in the aortic root axis between the sinotubular junction and VAJ,^6^ and dynamic changes in the aortic annulus level, area and shape during cardiac systole-diastole action,^5^ and size and shape progression at median follow-up.^7^^,^^8^

An interesting approach to the reimplantation procedure was presented recently in *JTCVS Techniques* by Woo and colleagues^1^ from Stanford. They embraced elements of remodeling by differing from the horizontal plane due to mitral curtain, bundle of His or VAJ, and close approximation to left-non, right-non, and left-right commissures, respectively.

On the other hand, it has been suggested that stabilizing the VBR is essential for a secure repair.^9^ To address this, different models of VBR stabilization have been developed. Among them, the internal annuloplasty, by definition, allows stabilization of the basal ring, certainly to reduce further dilatation, maintaining at the same time subcommissural triangles due to the crown-shaped 3-pointed structure of the ring, thus stabilizing the sinuses of Valsalva by virtue of coupling VAJ and VBR planes.^8^

The idea of combining both internal annuloplasty with the internal anatomic annuloplasty ring and external Dacron ring or prosthesis can be successfully adopted and is shown in Figure 1, as well as demonstrated by the tutorial,^10^ featuring the remodeling elements. This embraces concepts of VAJ remodeling and prosthesis trimming during reimplantation (Figure 2) and complete circular annuloplasty, both internally and externally,popularized by Nawaytou and colleagues^9^ and promoted by G.El Khoury^9^ and T. David.^11^

**Figure 1:**
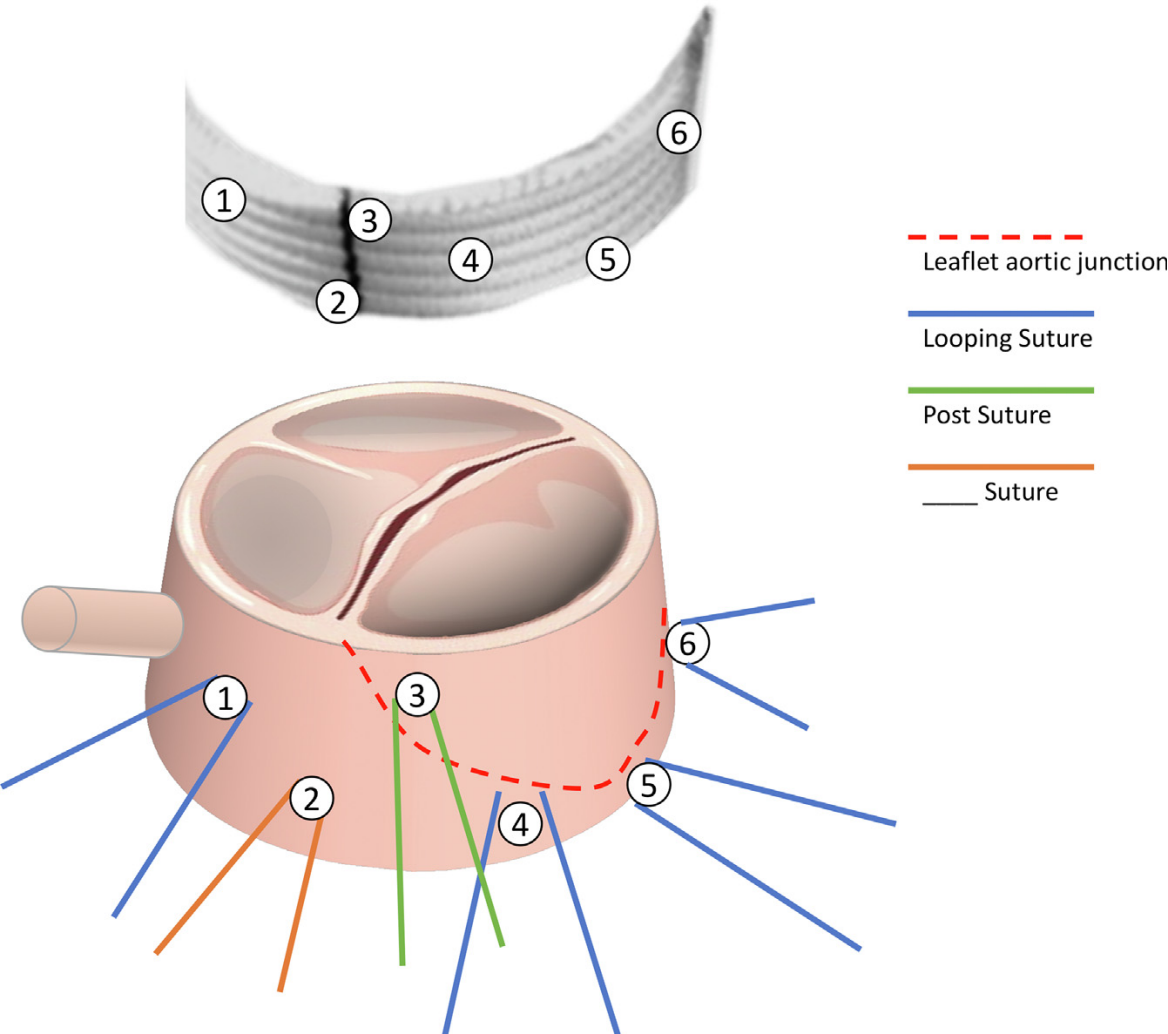
Concept of tailored external annuloplasty with anatomic inflow line.

**Figure 2:**
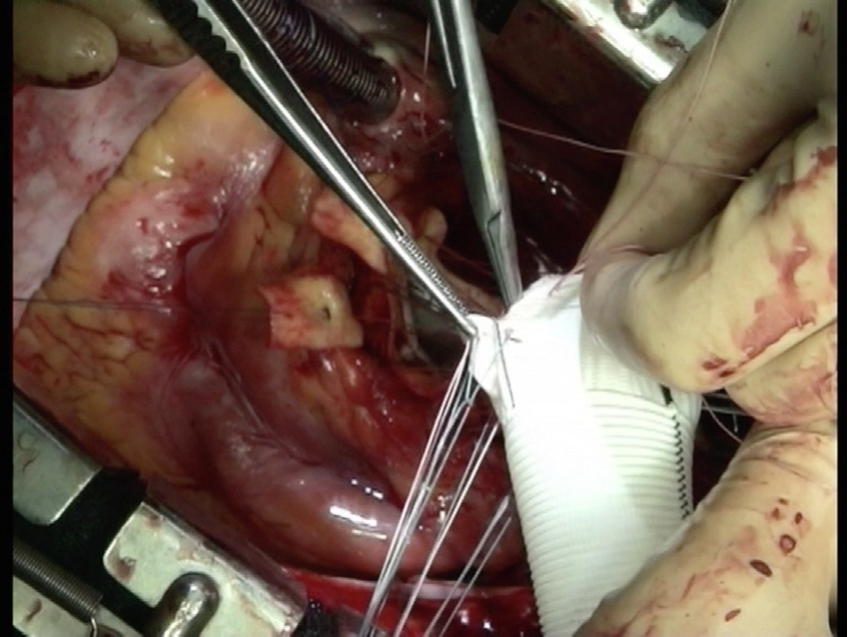
Tailored graft during reimplantation- author's technique.^12^

Stabilization and relative adjustment of VBR, VAJ, and sinotubular junction allow for physiological transmission of movements or cross-talk between the ventricle and the root with emphasis on the role of unobstructed root systolic root dilatation causing horizontal stretch and triangular shape of leaflets, according to Yacoub and colleagues.^8^ This implies tailoring the surgical approach to individual physiology translating into adopting different methods. It encourages us to remain open-minded and explore different techniques to improve the end result of aortic valve repair.

## Conflict of Interest Statement

Dr Jasinski: Medtronic and Corcym.

The *Journal* policy requires editors and reviewers to disclose conflicts of interest and to decline handling or reviewing manuscripts for which they may have a conflict of interest. The editors and reviewers of this article have no conflicts of interest.
